# A radial calibration window for analytical ultracentrifugation

**DOI:** 10.1371/journal.pone.0201529

**Published:** 2018-07-30

**Authors:** Thomas LeBrun, Peter Schuck, Ren Wei, Justine S. Yoon, Xianghui Dong, Nicole Y. Morgan, Jeffrey Fagan, Huaying Zhao

**Affiliations:** 1 Physical Measurement Laboratory, National Institute of Standards and Technology, Gaithersburg, Maryland, 20899, United States of America; 2 Dynamics of Macromolecular Assembly Section, Laboratory of Cellular Imaging and Macromolecular Biophysics, National Institute of Biomedical Imaging and Bioengineering, National Institutes of Health, Bethesda, Maryland, 20892, United States of America; 3 Biomedical Engineering and Physical Science Shared Resource, National Institute of Biomedical Imaging and Bioengineering, National Institutes of Health, Bethesda, Maryland, 20892, United States of America; 4 Materials Science and Engineering Division, National Institute of Standards and Technology, Gaithersburg, Maryland, 20899, United States of America; The Ohio State University, UNITED STATES

## Abstract

Analytical ultracentrifugation (AUC) is a first-principles based method for studying macromolecules and particles in solution by monitoring the evolution of their radial concentration distribution as a function of time in the presence of a high centrifugal field. In sedimentation velocity experiments, hydrodynamic properties relating to size, shape, density, and solvation of particles can be measured, at a high hydrodynamic resolution, on polydisperse samples. In a recent multilaboratory benchmark study including data from commercial analytical ultracentrifuges in 67 laboratories, the calibration accuracy of the radial dimension was found to be one of the dominant factors limiting the accuracy of AUC. In the present work, we develop an artifact consisting of an accurately calibrated reflective pattern lithographically deposited onto an AUC window. It serves as a reticle when scanned in AUC control experiments for absolute calibration of radial magnification. After analysis of the pitch between landmarks in scans using different optical systems, we estimate that the residual uncertainty in radial magnification after external calibration with the radial scale artifact is ≈0.2 %, of similar magnitude to other important contributions after external calibration such as the uncertainty in temperature and time. The previous multilaboratory study had found many instruments with errors in radial measurements of 1 % to 2 %, and a few instruments with errors in excess of 15 %, meaning that the use of the artifact developed here could reduce errors by 5-to 10-fold or more. Adoption of external radial calibration is thus an important factor for assuring accuracy in studies related to molecular hydrodynamics and particle size measurements by AUC.

## Introduction

Analytical ultracentrifugation (AUC) is a classical technique of physical chemistry [1], in which the temporal evolution or equilibrium concentration distribution of dissolved macromolecules or nanoparticles is optically measured in real-time under application of a centrifugal field [2]. This is achieved with an optical scanner or imaging system with light path perpendicular to the plane of rotation, which is synchronized with the revolution of the sample in a rotor spinning at ≈1 kHz, to measure the radial concentration profiles of particles and their changes with time. Its invention in the early 20^th^ century led to the birth of molecular biology and macromolecular sciences. Due to the simple physical concept, and the rich hydrodynamic and thermodynamic information it generates, modern AUC is widely used in many fields including biochemistry, structural and molecular biology, supramolecular and physical chemistry, biotechnology, and materials science.

Based on first principles, AUC allows measurement of molar mass, hydrodynamic radius, and interactions of macromolecules over a size range spanning three orders of magnitude in Stokes radius, even in the same experiment [3, 4]. It is also very flexible in sample concentration, which may span many orders of magnitude dependent on the particles of interest and choice of optical detection method, which in current commercial instruments could be fluorescence, absorbance, or refractometry.

Sedimentation velocity experiments (SV) observe the time-course of sedimentation and offer results with the highest precision, with the repeatability of sedimentation coefficients (‘s-values’) from replicate samples in the same run typically being on the order of 0.1 % [5]. The determination of absolute molar mass and frictional coefficients from sedimentation coefficients is dependent on the density contrast between particles and solvent; for proteins the relative uncertainty arising from the partial-specific volume typically amounts to ≈1 %, but this effect is less significant for particles of higher density in aqueous solutions. In comparison to other hydrodynamic methods, SV has the important advantages of allowing measurement in dilute solution in the absence of a matrix, and the ability to exquisitely detect and separate the effects of sample heterogeneity, such that very precise hydrodynamic parameters can be obtained even from samples of imperfect purity or which exhibit dispersity. In practice, therefore, SV-AUC is an excellent method—arguably the gold standard—for the study of hydrodynamic properties of macromolecules and nanoparticles. It has been used in many different types of applications. For example, in conjunction with theoretical structure-based hydrodynamic predictions [6–8], SV-AUC data provide insight on macromolecular structure, conformation, solvent interactions and/or particle composition [9]. Similarly, experimental hydrodynamic parameters from SV-AUC may be used as constraints in the prediction of solution structures, for example, in conjunction with small angle scattering [10]. Finally, data sets of translational friction coefficients from SV-AUC and rotational friction coefficients of particles of known structure can help to elucidate fundamental aspects of molecular hydrodynamics and hydration [11–13]. Furthermore, SV-AUC analysis plays an important role in regulatory applications, such as the characterization of biopharmaceuticals and potentially immunogenic trace protein aggregates [14] or nanomaterials [15].

For all of these applications, as well as those related to sedimentation equilibrium AUC, it is critical that AUC measurements provide highly accurate values. Errors in sedimentation coefficient strongly affect calculations of the biophysical attributes of the biomacromolecules under study, including shape estimation and the stoichiometry and geometry of supramolecular assemblies. A 5 % underestimate in sedimentation coefficient would make a spherical particle appear to have an ellipsoidal axial ratio of ≈2.1. Similarly, due to the 2/3-power scale relationship between mass and sedimentation coefficient of compact particles, when estimating the mass of particles of known shape, a 5 % error in the sedimentation coefficient would lead to an ambiguous or incorrect assignment of protein oligomers of hexamers and higher assemblies. Lastly, even stronger error amplification can occur in cases for which the buoyant molar mass implied by hydrodynamic measurements is used to estimate the chemical composition of a particle [16, 17].

With typically excellent statistical properties of data acquisition and highly developed computational data analysis [18, 19], the accuracy in AUC ultimately rests on the calibration accuracy of the instrument. In a recent series of studies, improved calibration procedures have been developed in order to compensate for unexpectedly large systematic errors that were discovered to occur in the reported elapsed time after start of rotation [20, 21], the rotor temperature [21–23], and radial calibration of AUC data [21]. An analysis of the impact of radial calibration errors showed that the radial magnification error is the dominant term, approximately an order of magnitude more important than translational errors in the radius scale [21, 24, 25]. In a multilaboratory benchmark study [25], sedimentation coefficients of a reference sample measured using steel calibration masks in 79 instruments in 67 laboratories revealed radial magnification errors after using the manufacturer’s calibration of ≈ 1 % ± 3 % (mean and standard deviation), with several outlier instruments determined to have magnification errors more than 15 % [25]. In a minority of instruments, non-linear distortions across the radial measurement range were also observed. It is important to note that these calibration errors are not easily discerned from the collected data, and that systematic calibration errors in an instrument will not be revealed through repeated experiments. Calibration with external standards is therefore essential for accurate quantitative measurements with the AUC instruments [25]. In the multilaboratory study, after measurements made with the external calibration standards to correct for errors in time, temperature, and radial magnification, the measured sedimentation coefficient values for a common sample exhibited a 6-fold reduced standard deviation and 7-fold reduced range, with a final standard deviation for the whole population of instruments of 0.7 %. This demonstrates that these calibrations are both critically important and highly effective in achieving reliable quantitative results from AUC analysis.

Methods for calibrating scan time [20] and rotor temperature [21–23], which were applied and validated in the multilaboratory study [25], can be easily implemented in most laboratories using existing or readily obtainable equipment. However, a readily available external radial magnification standard is still lacking. Previous work on calibrating the radial dimension used a custom-fabricated patterned steel mask sandwiched between windows in a cell assembly, to produce a light transmission pattern exhibiting light-to-dark transitions at well-known radial intervals [21]. Here, we describe an improved radial magnification calibration artifact that has the following advantages: (1) The pitch (i.e., the distance between radial features) is calibrated traceably to the International System of Units (SI) unit of length, the meter at the U.S. National Institute of Standards and Technology (NIST); (2) The mask is lithographically patterned onto a sapphire substrate that can serve as an AUC sample cell window, simplifying sample cell assembly and offering improved mechanical rigidity of the scale against deformations at high centrifugal fields; (3) The thin, opaque chrome pattern on the window functions as a more ideal mask, giving less scattered light and edge roughness; (4) An offset dual pattern produces a larger number of data points, and allows for detection and correction of rotational misalignment of the mask; and (5) The artifact is suitable to be manufactured at a sufficient scale to serve as a Standard Reference Material (SRM) distributed by NIST. In the present communication, we characterize a prototype of the artifact, and examine the performance of prototype artifacts in the current commercial analytical ultracentrifuge, using a new software package, “MARC” (Mask Analysis for Radial Calibration), that we developed for the analysis of radial calibration scans. It is freely available as an executable for Windows operating systems, and can be downloaded from sedfitsedphat.nibib.nih.gov.

## Materials and methods

### Calibration window

**Fig 1:**
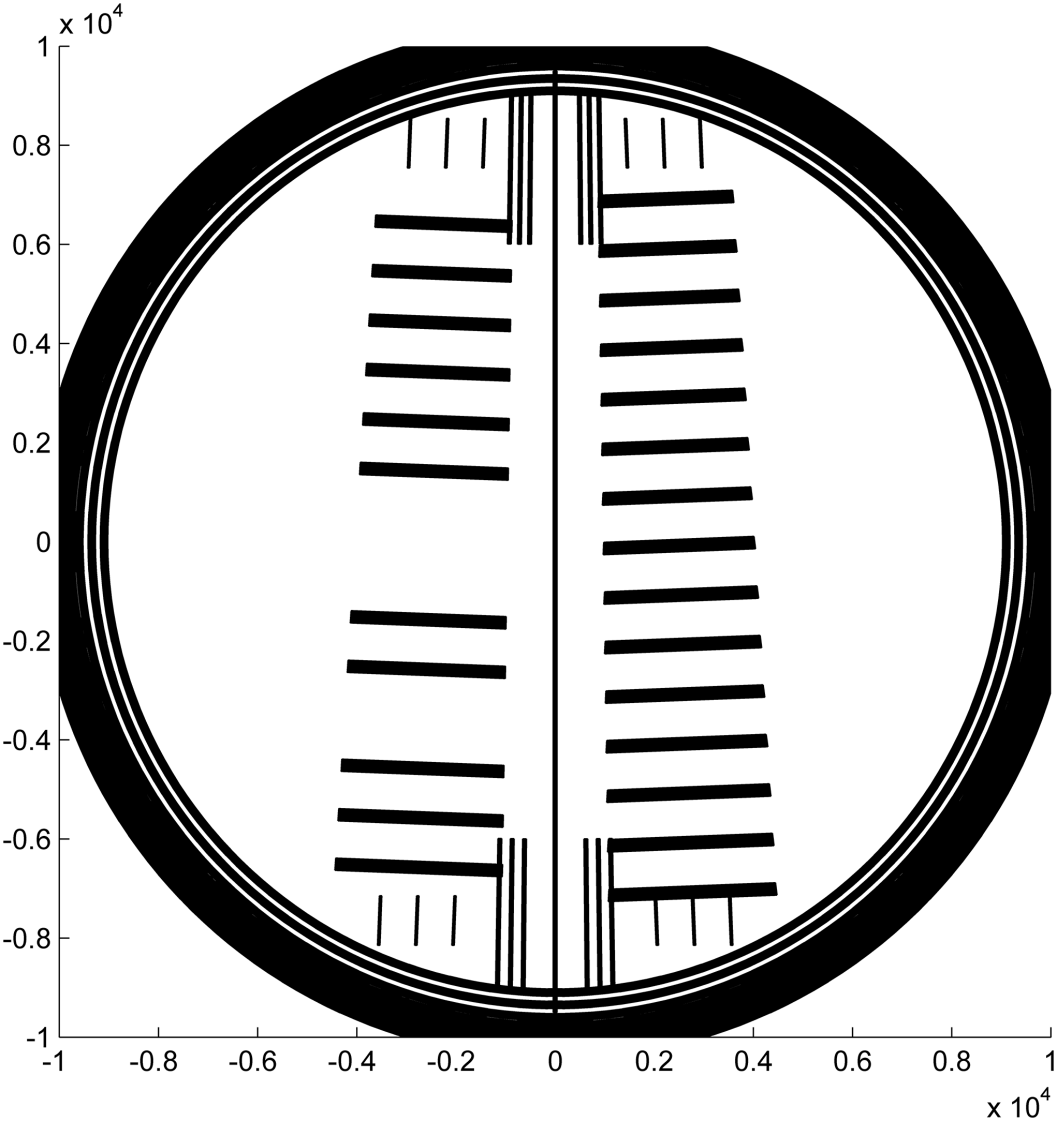
Drawing of the mask design. Two scales (series of lines) are perpendicular to the radius from the center of rotation once placed in the rotor hole of the analytical ultracentrifuge. The sample side (right) and reference side (left) have the same pitch of 1 mm, but are shifted by half the pitch. A long central line and 12 short lines near the center are added as a visual guide for mounting the window into the cell assembly, to facilitate angular alignment relative to the middle divider of a centerpiece. Three short lines above and below each scale are features recognized when calibrating the artifacts for certification and encompass the angular range over which the scale pitch is determined. The units of the axis drawn are micrometers.

A pattern was designed as shown in Fig 1, with two sets of line scales (running top to bottom in the figure), each aligned radially with respect to the center of AUC rotation. Each scale nominally consists of a series of 0.25 mm-wide lines separated by 0.75 mm to give a pitch of 1 mm. The two scales cover the sample sector (right) and the reference sector (left) of a standard sample assembly. Both sectors have nominally the same pitch but the scales are offset by 0.5 mm radially so that the line locations are spatially interleaved at the detector. In this design the comparison of the line locations from both scales can reveal rotational misalignment of the window through displacement of the two scales relative to each other. The prototypes used in the present study were designed with linewidths of 0.228 mm. (The linewidths as printed were not measured because they are not necessary for pitch calibration and because proper assessment of linewidth uncertainty requires extensive modeling beyond that necessary for pitch calibration). The lines are perpendicular to the radius at the center of each sector, in order to minimize errors arising from angular offset in scanning (i.e., in the terminology of the AUC user interface, from imperfections in the ‘delay calibration’ of the rotor angle associated with optical alignment of each sample position during rotation). Patterning the scale lines as arcs is not necessary; the angular span of the sectors as measured from the center of rotation is approximately 2°, such that the corresponding maximum error due to scanning angle variation is only approximately 0.015 % (calculated as 1-cos(2°/2)). Three lines of the scale for the reference sector are omitted to allow for unambiguous identification of the window orientation from the scan data through both sectors. A center line facilitates visual inspection of the rotational alignment when mounting the window in the window holder, by comparison with the central divider on a double sector centerpiece.

Even with features to guide alignment of the artifact to the centerpiece, errors could arise from rotational misalignment of the cell assembly in the rotor, and/or of the window within the window holder. Rotational misalignment of the cell housing was previously estimated to be usually less than 0.5° based on scribe lines and grooves in the aluminum barrel and rotor [26, 27]. In either case, due to the offset line design of the artifact, the total rotational misalignment would cause a shift in the imaged positions of the edges in the reference beam sector relative to those in the sample sector, which would not be symmetrical anymore but appear systematically shifted by ≈10 % of the nominal edge distance with a 0.5° rotation error. Thus, the detection of a phase shift of the measured edges allows for calculation of the rotational angle of the window, and application of correction factors to accurately determine the measured pitch and radial magnification of the optical system.

Windows were fabricated from sapphire (Meller Optics Inc., Providence RI) to give blanks of 19.05 mm (0.75”) diameter and 5.08 mm (0.2”) thickness with the c-axis parallel and in the plane of the faces. CAD data were supplied for lithographic patterning and deposition of absorptive ‘blue-chrome-blue’, with image centralization of ± 50 *μ*m, by Applied Image Inc. (Rochester NY). Blue chrome is less reflective due to an oxide coating approximately one quarter wavelength thick. The designation”blue-chrome-blue” refers to chrome that has an oxide coating on both sides. It was chosen to reduce optical scattering from a highly reflective substrate, and on the assumption that the oxide layer confers greater chemical resistance and stability than metallic chrome.

### Measurement and accuracy

Dimensional measurements of the scale pitch, scale angle with respect to the center line, and radial offset with respect to the center of rotation were performed on a Nikon optical coordinate measuring machine (OCMM) (model number VMR-6555). The Nikon OCMM was calibrated and error mapped using a 2D grid plate calibrated on the NIST Line Scale Interferometer [28], which provides direct traceability to the meter with an expanded uncertainty of less than 10 nm (k = 2) for a scale length of 1 mm. The expanded measurement uncertainty of the Nikon is a few hundred nanometers and details of the artifact production and calibration will be published when the artifacts are issued. The target uncertainty for the artifact pitch was selected to be approximately 1 *μ*m, to provide a standard that fully satisfies the requirements of AUC without unduly increasing cost. Unless otherwise noted, the reported uncertainty is one standard deviation, which corresponds to a coverage factor of one.

### Analytical ultracentrifugation

Analytical ultracentrifugation experiments were carried out in a ProteomeLab Optima XLA/I (Beckman Coulter, Indianapolis, IN) at 5236 rad/s (50,000 rpm) and 20.0°C using absorbance or interference detection. Alternatively, the fluorescence detection system (FDS, Aviv Biomedical Inc., Lakewood, NJ) was used with excitation at 488 nm (10 mW power). In either circumstance, the calibration window was mounted in a standard cell assembly with a 12 mm carbon-filled epoxy centerpiece, or elevated to the desired height in the absence of a centerpiece with the aid of spacer rings. Prior to the experiments with any of the systems, unless mentioned otherwise, radial calibration was carried out according to the manufacturer’s instructions. The absorbance system was used in the intensity mode to acquire the transmitted light for the sample and reference sector separately, stepping with a radial resolution of 0.01 mm. Fluorescence data were collected with 0.01 mM fluorescein (in a buffer of 10 mM Tris, pH 7.8, 100 mM KCl) in a 12 mm centerpiece. Raw scan data can be found in the Supporting Information S1 Dataset.

### Analysis of scan data

A software program MARC was developed to facilitate the determination of radial calibration correction factors from experimental scan data. For the absorbance system, files containing scans of the transmitted light intensity measured at both sample and reference side were loaded. Maxima and minima of the radial intensity derivatives, calculated using a Savitzky-Golay filter, were automatically selected as markers for the radial position of the upper and lower edges of each line, *u*_*i*_ and *l*_*i*_. The precision of each edge position is limited by the fixed 10 *μ*m interval of reported scan data; a standard deviation of 6.8 *μ*m for equivalent positions was determined from the analysis of 10 replicate scan files. The same procedure was carried out for the fluorescence data. Analogous reference points in interference optical data can be selected either manually from the graph by identifying the periodic pattern in the fringe shift data at the edges, or by automatically exploiting a feature of the radial derivative of the fringe shift data. A global linear fit *u*_*i*_ = *u*_0*s*_ + *i* × *p*_*s*_ and *l*_*i*_ = *l*_0*s*_ + *i* × *p*_*s*_ was used to determine the apparent pitch of the sample side, *p*_*s*_ and the radial offset *u*_0*s*_. Separately, the apparent pitch and offset were determined analogously for the reference side, *p*_*r*_ and *u*_0,*r*_.

By design, the pitch on both sides is identical and they can be averaged to determine the measured apparent pitch *p*_app_ := (*p*_*r*_ + *p*_*s*_)/2. However, the patterns on the sample and reference side are interdigitated, or offset, by one half period as they scan across the detector. The design difference between the offsets of the pattern is *u*_0*s*_ − *u*_0*r*_ = *l*_0*s*_ − *l*_0*r*_ = *p*_app_/2. However, this does not hold if the window is rotated by an angle *α* when the cell is loaded in the instrument; in this case an additional displacement Δ*r*_*α*_ of the patterns in the sample *vs*. reference sector is obtained, which can be measured both from upper or lower line edges, or Δ*r*_*α*_ = [(*u*_0*s*_ − *u*_0*r*_) + (*l*_0*s*_ − *l*_0*r*_)]/2 − *p*_app_/2. From the measured Δ*r*_*α*_ and the known angular separation of the bar centers *β* = 2 × 2.2° between sample and reference side, we can determine the angle in good approximation as *tan*(*α*) = Δ*r*_*α*_/(*r*_*m*_ × *sin*(*β*)), where *r*_*m*_ is the middle of the window at 65 mm. (For the small angles encountered here, *α*/Δ*r_α_* ≈ 11.5°/mm).

A radial correction factor *R* can be determined as the ratio between the ‘true’ pitch of the artifact along the line of measurement *p*^0^/*cos*(*α*) (where *p*^0^ is the calibrated pitch of the scale), and the experimental apparent pitch *p*_app_ in units of the instrument: *R* = *p*^0^/(*p*_app_ × *cos*(*α*)). Thus, multiplication of apparent radial positions of the experimental scans with the radial correction factor *R* will bring radial scan files on the absolute radial scale of the reference artifact. In a first-order approximation, this causes a relative change to *s*-values measured on the apparent scale by the factor (*R* − 1), and allows us to determine the relative error of the sedimentation coefficient measurement on the apparent scale Δ*s*/*s* = 1 − *R*.

## Results

### The artifact

**Fig 2:**
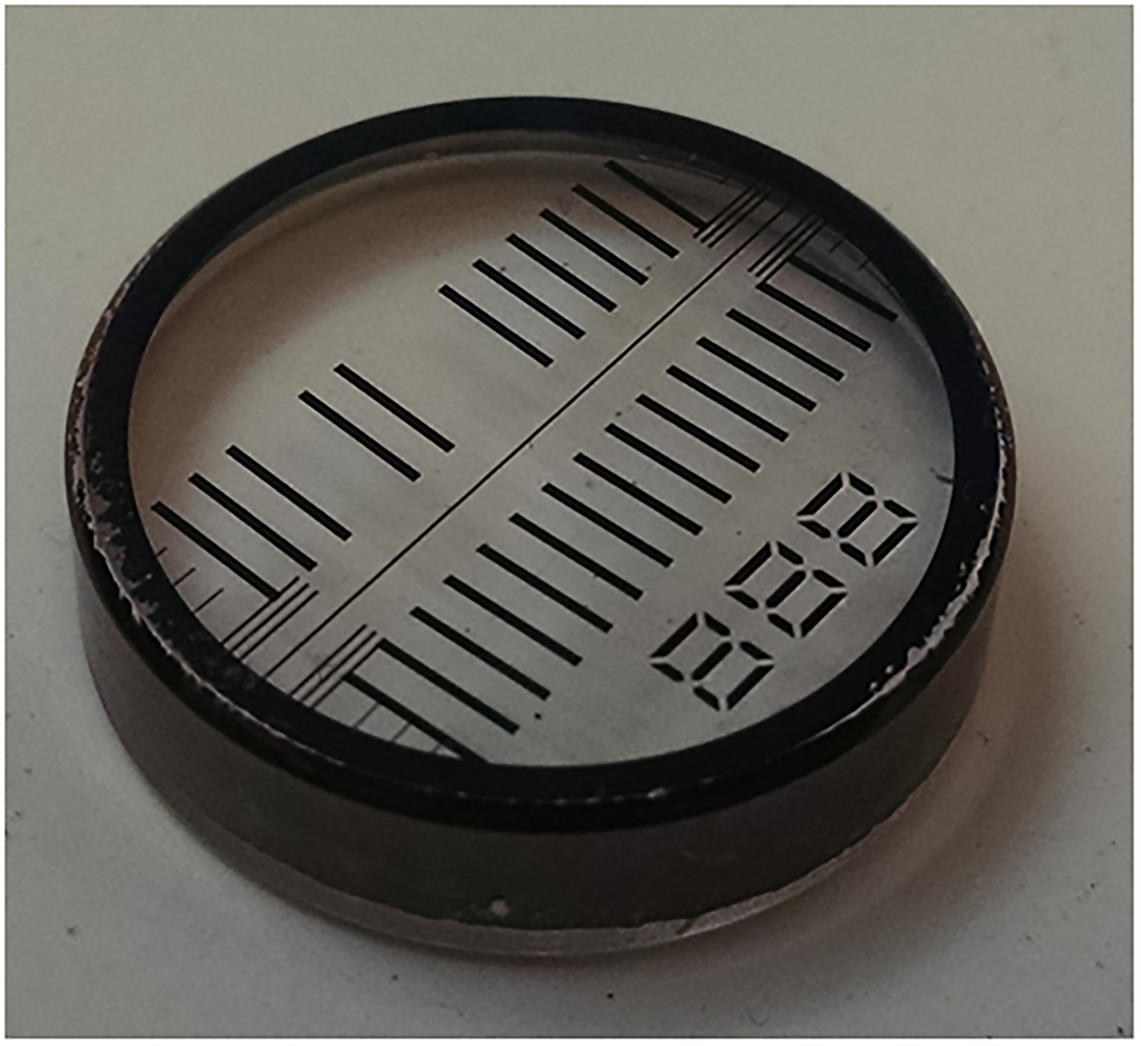
Picture of the radial scale artifact. The artifact is comprised of a lithographic chrome mask deposited on a sapphire substrate (a standard 3/4 inch diameter AUC window). The average pitch was measured to be 0.999748 mm.

In SV, the sedimentation coefficients are typically measured across a distance of approximately 10 mm. We set the target maximal error from radial calibration at 0.1 %, which is at the limit for repeatability of sedimentation coefficients (*s*-values) and below errors from uncertainties in the temperature calibration [21]. This translates to a target maximum error across the ≈10 mm window of 10 *μ*m, and a target maximum error of 1 *μ*m on the 1 mm pitch. Lithographic masks are typically expected to be much more accurate. A picture of a prototype is shown in Fig 2. The design pitch is 1 mm, and the average measured pitch for this particular artifact is about 252 nm shorter (*p*^0^ = 0.999748 mm) with an expanded uncertainty for the pitch of 500 nm (k = 2). The measured value was used for the analysis below. The deviation of the pitch of the artifact from the nominal design value reflects manufacturing spread. The relative size of the manufacturing spread for the final production run of artifacts versus the target uncertainty of 1 *μ*m will be used to determine the appropriate calibration and use of the artifacts.

It is important to note that the optical systems for each of the three AUC measurement systems tested in this contribution are distinct, and thus the radial magnification calibration for each system is independent of the others. We thus address the calibration and results of each optical system in sequential fashion.

### Radial calibration in the absorbance optical system

**Fig 3:**
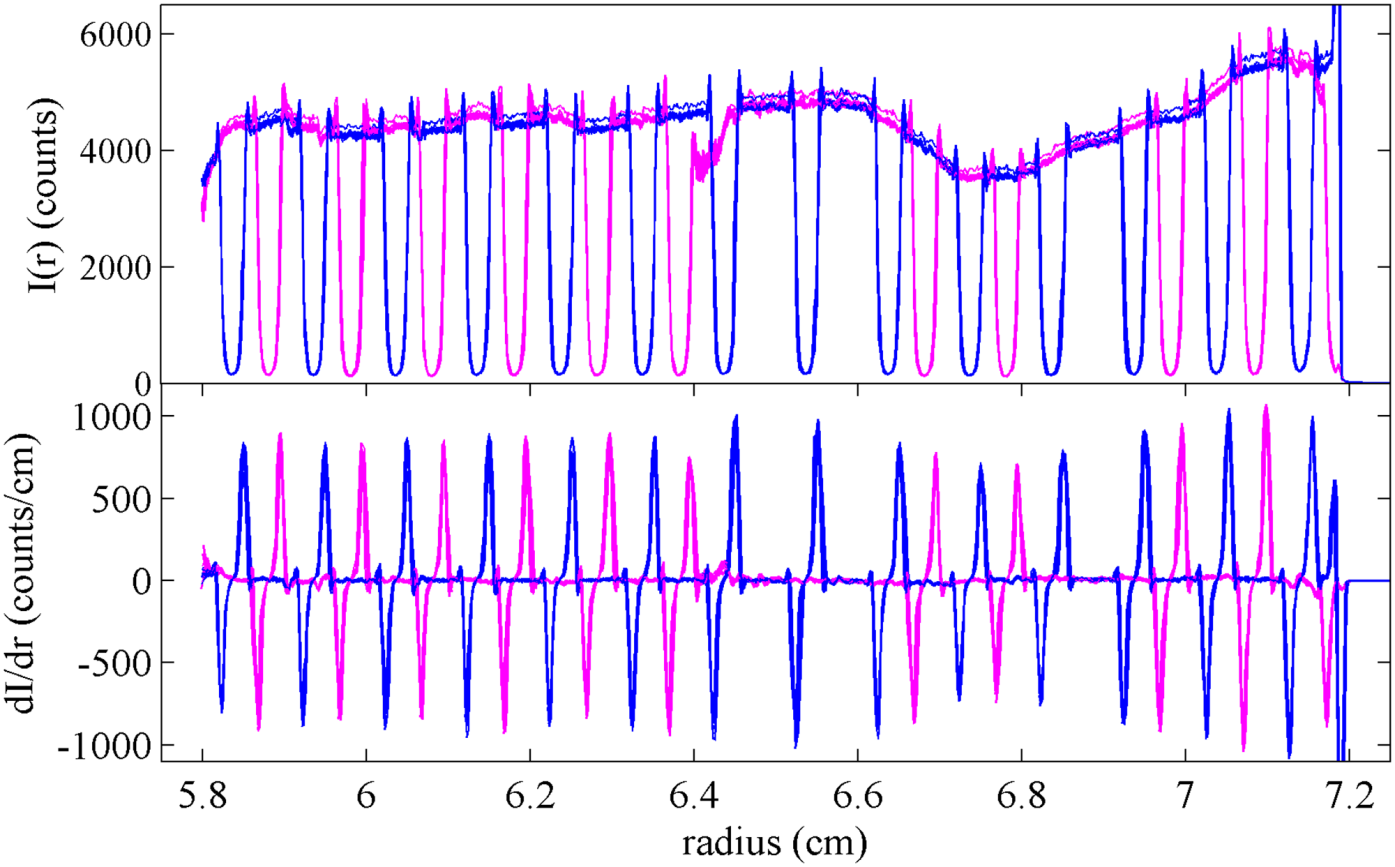
Radial scans of transmitted intensities. Superposition of 10 intensity scans of the artifact using the absorbance optics at 280 nm in stepping mode with radial resolution of 0.001 cm, showing the signal of the sample side (blue) and reference side (magenta). The top panel shows the raw data, and the bottom panel shows a derivative plot (calculated with Savitzky-Golay filter [29] with frame length of 7 and polynomial order of 2). The intensity of the transmitted light varies slightly and with low spatial frequency due to variations of the local photocathode sensitivity.

We first examined the performance of the artifact in the SV using the absorbance optical system. Fig 3 shows a superposition of radial profiles of the transmitted light intensity measured with the artifact mounted into a regular cell assembly with a 12 mm centerpiece, in a configuration where the mask is in the upper window closer to the light source and facing the centerpiece. Except for slight and inconsequential variations in the total intensity, the scans are highly reproducible, and their derivative provides a convenient estimate of the edge positions within the visible radial range.

Even though it is unclear whether the maximum slope precisely reflects the radial position of an edge, it is reasonable to assume any offset will be the same for all equivalent edges along the radial scan. Although a more detailed interpretation of the shape of the light-to-shadow transition is possible, it would make the analysis dependent on the detailed optical alignment of the particular instrument. The previous multilaboratory study [25] using a steel mask found significant instrument-to-instrument variation in the detailed shape of light-to-shadow transitions. The new artifact used here may reduce the variations seen between instruments, because the thinness of this pattern should reduce edge scatter relative to the steel mask. Nevertheless, since a more detailed analysis is unnecessary for the uncertainty required here, it is preferable at present to solely rely on the pitch of the pattern for equivalent edges.

**Fig 4:**
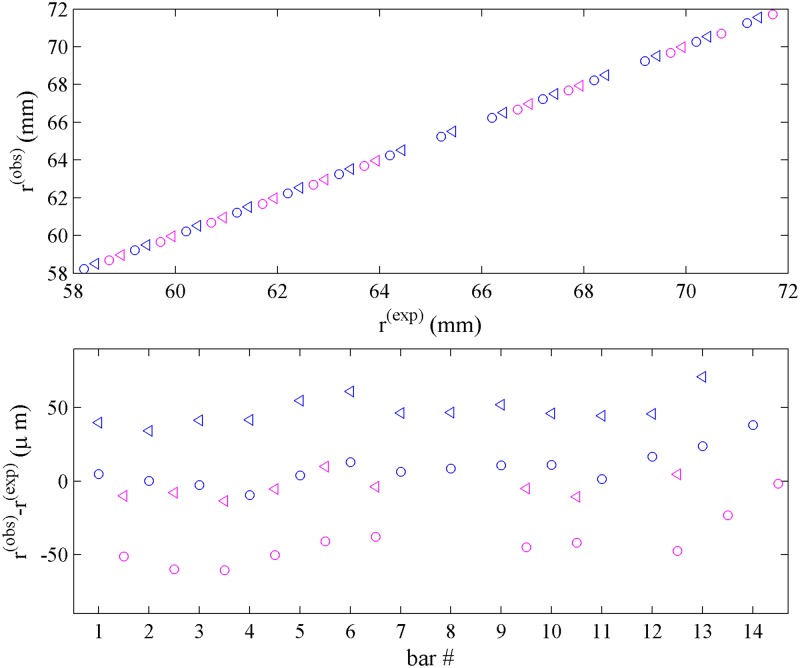
Analysis of the intensity profiles of Fig 3. Top: Points of maximum slope in the transmitted signal for light-to-shadow transitions (circle) and shadow-to-light transition (triangle) of each bar in the sample side (blue) and reference side (magenta), *r*^(*obs*)^, plotted against the ideally expected position of the line edges, r(exp), given the known pitch of *p*^0^ = 0.999748 mm and known line width of 0.228 mm. Due to the unknown overall radial position of the mask, the expected positions have the same arbitrary offset. Bottom: Difference between the radii of the measured transitions and the expected location of the edges of the mask. Slopes in this plot visually highlight differences in the pitch. Irrespective of the pitch, for a mask with perfect rotational alignment, the difference between measured and ideally expected edge positions would be the same for corresponding edges in the reference and sample side, i.e., equivalent blue and magenta symbols would not be offset. The observed (≈50 *μ*m) displacement between sample and reference transitions seen from the vertical shift between blue and magenta symbols in the bottom panel reveal rotational misalignment, here corresponding to 0.51°. This rotation will also change the measured pitch along the line of measurement very slightly, making it larger by *p*^0^/*cos*(*α*) − *p*^0^, which here amounts to 0.04 *μ*m. The displacement between triangles and circles reflects the difference between observed and designed linewidth, a feature not utilized in the analysis due to its susceptibility to optical misalignment and the detailed shape of the measured transitions.

The measured transitions are shown in Fig 4. The top panel displays the radial positions for each line, while the bottom panel shows the difference between the measured and expected position of each edge, *modulo* an overall constant offset due to the unknown absolute radial position of the entire mask. Differences between the measured pitch of the transitions and the known pitch of the lines appear in this representation as slopes of equivalent symbols. In addition, the difference between apparent left and right edge positions of each line is depicted by the displacement between circles and triangles of each color. As expected, the observed value for the apparent line width from transition points (0.27 mm) is slightly larger than the known line width (0.228 mm). Further, in the bottom panel a consistent off-center displacement of the transitions comparing sample and reference side can be recognized from the displacement between equivalent blue and magenta symbols. From the average displacement of the patterns between the sample and reference side a rotation angle of 0.51° is calculated, and from the standard deviation of the displacement data within sets of equivalent edges of ≈8 *μ*m, we estimate a statistical error in the angular rotation of ≈0.1°. The impact of this rotation on the pitch in the line of measurement is only ≈0.004 % and therefore negligible. More conservatively assuming that the precision of the measured transition radii is on the order of 10 *μ*m, for the pattern of radial points shown in Fig 4, the statistical error of transition points propagates into a statistical error of the measured pitch of 0.04 % [30].

**Fig 5:**
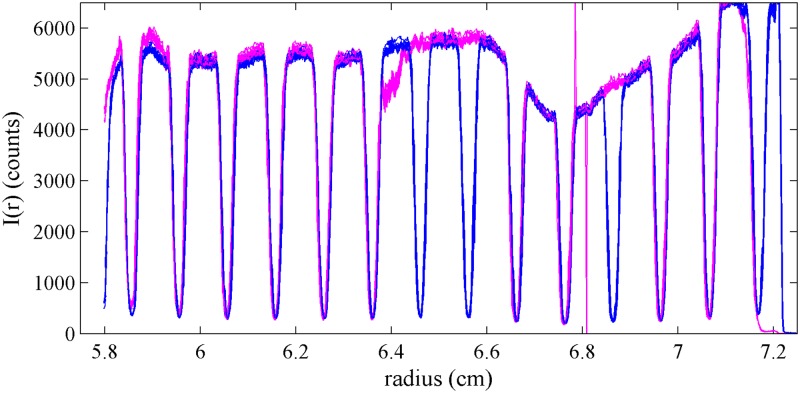
Intensity profiles of a rotated artifact. Intensity profiles acquired in the absorbance optics after intentional rotation of the artifact by 5.7°. Due to the rotation, the pattern from the sample sector (blue) and the reference sector (magenta) almost superimpose, rather than showing the half pitch offset expected at perfect rotational alignment.

A more extreme example of the effects of rotational misalignment of the window is shown in Fig 5, where the artifact was intentionally rotated. In this case, the radial positions of the lines appear to coincide in the sample and reference sectors. Here, the rotation by 5.7° increases the pitch along the line of measurement by 0.5 %, which is significant. Thus, measurement of the rotation angle is useful to prevent rotational alignment errors from propagating into radial calibration errors. We also carried out experiments with the mask located in different positions along the height of the cell assembly (i.e., along the light path of the detection), without observing discernable effects on the intensity profiles (data not shown).

To examine the precision and repeatability of the calibration, we carried out a series of experiments in the same instrument. Repeat experiments exhibited differences of the obtained correction factors Δ*s*/*s*, on average, of 0.15 %. This variation is ≈4-fold larger than the expected statistical error propagated from the error in the individual transition points. We have previously observed that manufacturer’s radial calibration errors are dependent on the particular instrument but stay constant for long periods of time [21, 25]. However, at each instance of an AUC experiment, the commercial instrument will carry out some initialization operation of the optical system, including the ‘delay calibration’ determining the angular position of the samples in the rotor relative to a magnetic timing pulse in the rotor, and possibly other steps that could contribute to run-to-run variation for the identical mask.

Detailed results obtained in repeat experiments varying the rotation angle and placement of the same prototype mask within the cell assembly are provided in Table 1. The first three rows of data show the effect of increasing angular misalignment in one particular instrument (Larry). Without instrument re-calibration in between experiments, the error in the radial scale for this instrument should be unchanged. Focusing first on the absorbance data, the Δ*s*/*s*-values obtained for three rotations change by 0.25 %, showing satisfactory compensation of the effect of rotation on the pitch in the line of measurement. The next two rows of Table 1 show the effect of varying placement of the artifact in the cell, exhibiting similar small variation. The last two rows of data in Table 1 are experiments using a different instrument (Shemp) observed to have a larger error than Larry, as reflected in the Δ*s*/*s*-values of ≈2.4 %. Here, we also compared the lithographically patterned masks with the steel mask used previously for radial calibration [21, 25], and note good agreement, even though the steel mask is less accurate and does not allow to correct for rotational misalignment.

**Table 1:** Calibration constants determined in different configurations.

instrument	placement	absorbance system			interference system			fluorescence system		
		*α* (°)	*R*	Δ*s*/*s* (%)	*α* (°)	*R*	Δ*s*/*s* (%)	*α* (°)	*R*	Δ*s*/*s* (%)
Larry	up/outside	0.51	1.0005	-0.05	0.16	1.0015	-0.15			
	up/outside	2.40	0.9988	0.12	2.04	0.9996	0.04			
	up/outside	5.83	0.9980	0.20	5.50	0.9986	0.14			
	up/inside	2.65	1.0025	-0.25	3.01	1.0008	-0.08			
	down/outside	0.63	0.9999	0.01	0.70	0.9989	0.11			
Shemp	up/outside	0.88	1.0239	-2.39	0.53	1.0082	-0.82			
	center steel mask*		1.0286	-2.86		1.0083	-0.83			
Moe	up/inside							2.30	0.9979	0.21

Experiments were carried out sequentially without invoking instrument radial calibration functions in between. The placement ‘up’ and ‘down’ refers to the use of the artifact as the window preceding the centerpiece or following the centerpiece in the light path; ‘outside’ and ‘inside’ refers to whether the lithographic layer faces the vacuum or the centerpiece. The corrections to the s-values (Δ*s*/*s* = 1 − *R*) show the magnitude of error that would be expected without calibration.

* For comparison, the steel mask introduced in previous external calibration work was mounted in the middle of the cell [21].

### Radial calibration in the Rayleigh interferometry system

**Fig 6:**
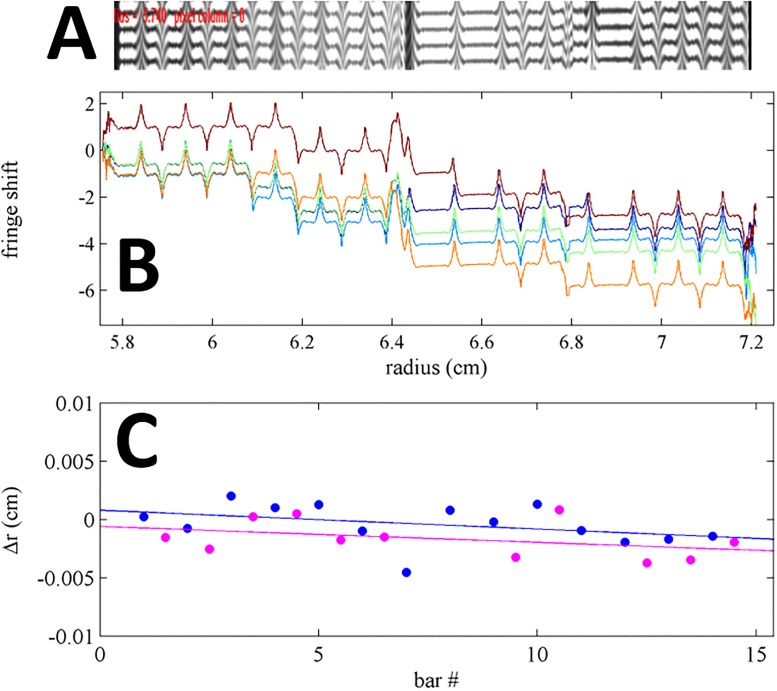
Calibration data in the Rayleigh interference optical system. (A) Screenshot of the AUC interference camera image from the artifact (expanded to the same radial scale as in B). Due to diffraction effects in this instrument, the regions of the bars are still illuminated with strongly sloping fringe shifts at each edge, forming a maximum or minimum for sample or reference side, respectively. (B) Overlay of 5 fringe shift profiles produced by the instrument software from the camera data, sequentially acquired in the same run. The discontinuity of fringes at the center of each bar can cause integral fringe shifts in the reported data. Peak locations were taken as landmark for the determination of pitch. (C) Difference between landmarks for each bar of the mask in the sample (blue circles) and reference sectors (magenta circles), and the calculated position based on the known pitch of the artifact. Best-fit linear regressions are shown as solid lines; their average slope corresponds to the instrument calibration errors of the fringe shift data, and their displacement reveals rotational misalignment of the artifact.

Next we studied the performance of the lithographic mask in the Rayleigh interferometry detection system. It would be most desirable to carry out the calibration directly using images from the camera, which can be exposed with light traversing through the different sectors separately or jointly, thus allowing the mask to create various patterns of uniform dark and light zones and zones of interference [2]. However, the commercial system provides no convenient access to the raw images, and the default image processing algorithm that creates the radial fringe shift datasets has to be used for calibration. This provides a single radial profile that reflects fringe shifts from zones where light from both sectors can interfere, thus reflecting edges from both sector and sample side at once (Fig 6A). Ideally, 0.25 mm stretches of fringe data will alternate with 0.25 mm stretches of shadow, and fringe data will alternate in length when subject to rotational misalignment. The sample and reference side can be distinguished based on the lack of bars in the reference side.

In practice, dependent on the position of the artifact and exposure parameters, the dark region can still record some refracted light causing fringes, but is often subject to integral discontinuities (Fig 6B). From our experience with the multi-laboratory study, we expected the detailed appearance of the edges to be highly variable from instrument to instrument due to differences in the optical alignment. This was also observed in the comparison of fringe shift data when using the artifact in different instruments in our laboratory. However, the features are highly consistent from edge to edge along a single scan, after allowing for baseline offsets. Therefore, a similar situation arises as in the absorbance system, where a detailed physical model of the edge shape is not possible, and not necessary for radial calibration, as the pitch between equivalent features can be measured accurately. In the present work, to aid in the manual selection of the radial reference points, graphic displays of raw data scans or derivative plots were used. An example of interference fringe shift data from the artifact is shown in Fig 6B, from an instrument for which the peak fringes appear from refraction at the edges and provided a convenient landmark for reference points.

Quantitatively, the pitch between the measured radial positions of the landmarks can be compared with the known pitch of the artifacts, and a systematic slope in their difference determines the radial magnification correction factor (Fig 6C). In a series of experiments applying the artifact in different positions, and with different rotational mis-alignments, consistent radial correction factors were obtained, with a standard deviation 0.12 %, as judged from the values listed in Table 1 (it should be noted that the radial scale of absorbance and interference systems are independent and different). Similar to the results with the absorbance system, consistent results are obtained with the lithographic artifact and the previously used steel mask [21, 25].

The results for the absorbance and interference optical systems listed in Table 1 in each row are performed quasi-simultaneously without moving the artifact, just acquiring data with the different optical systems. Therefore, even though the radial scales of the absorbance and interference optical detection systems are independent, leading to different radial magnification calibration errors, the measurements of window rotational misalignment should coincide. Comparing the data from the two detection systems, angular alignment estimates agree better than 0.4°. (This amounts to about 20 % of the rotational angle, except for very small misalignment where the difference in *α* can be large but the scale change is negligible). Interestingly, for up/out, *α* is consistently larger for absorbance by about 0.34°. This could be due to misalignment of the optical axes of the two systems, and is supported by the fact that the sign and magnitude of the difference changes for the up/inside down/out configurations. Unfortunately, a full analysis of the optical alignment of the two systems is outside the scope of the present work.

With the interference optical detection system, an additional test for the performance of the calibration artifact is possible, due to the manual calibration procedure required for this detector. In the manufacturer’s calibration protocol for the interference optics, the operator must identify the edges of two holes in two masks set into the standard counterbalance. Setting these edges based on the camera image assigns the specified radii values from the manufacturer to these edges in the software. The assignment of these radii is a well-known source of operator error, but in the present study also offers a simple manner of causing intentional mis-calibration. For this reason, two sets of experiments were performed with the artifact in place to test performance of the artifact generated radial magnification calibration in comparison to the manufacturer specified calibration procedure. Data were acquired first with the conventional manual calibration, and then again after introduction of a well-known calibration error in marking the position of the holes in the masks in the counterbalance incorrectly. The analysis of the artifact should allow to correctly quantify the magnitude of mis-calibration in the comparison of calibration factors from the two scenarios. The intentional mis-calibration was carried out by selecting as landmarks in the camera the onset of complete shadow in the reference hole, rather than the onset of fringes—this reflects a common user error—which artificially stretches the radial scale of recorded data by 3.58 %. After analysis of the scans from the artifact, both scenarios show a difference in radial correction factors by 3.39 %. Thus, the external calibration with the analysis of scans from the lithographic mask can detect and correct for the mis-calibration to within 0.19 %.

### Radial calibration in the fluorescence optical system

**Fig 7:**
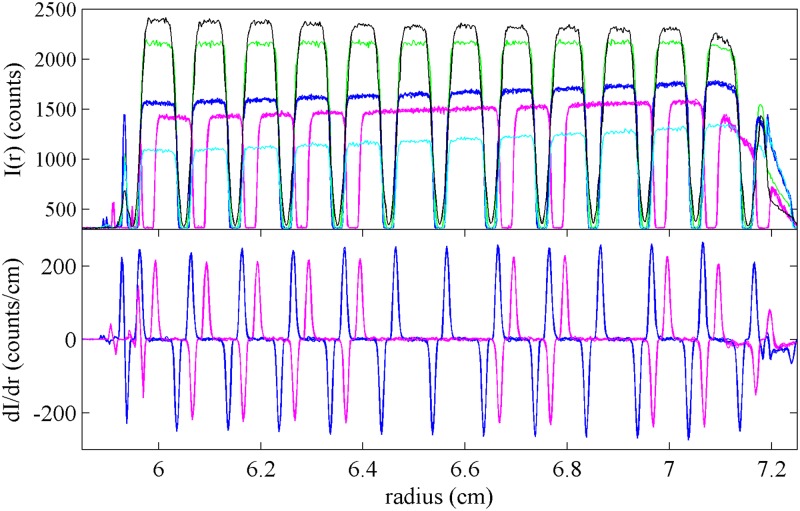
Fluorescence data using the artifact. Superposition of fluorescence scans taken with the lithography pattern facing the centerpiece containing a fluorescein solution. (Top) Shown are scans from sample sector at focal depth of 3955 *μ*m (black), 2697 *μ*m (green), 2196 *μ*m (blue, superposition of 4 replicates), and 1695 *μ*m (cyan), and from the reference sector at focal depth of 2196 *μ*m (magenta, superposition of 4 replicates). (Bottom) Superposition of derivatives of all traces acquired at a focal depth of 2196 *μ*m for sample (blue) and reference sector (magenta).

The fluorescence optical detector resembles a confocal microscope radially scanning the cell assembly from the top with a focused excitation beam, not observing light transmission as in the absorbance and interference detector. Therefore, for the application of the external calibration to the fluorescence optical data, the artifact was positioned into the upper window with the mask facing onto the centerpiece containing a fluorescein solution. The shape of the fluorescence scans depends on the focal depth, which in this optical system is adjustable over a wide range (Fig 7). Usually, to limit inner filter effects, focal depths of ≤4 mm are used, but due to the convergent light path of excitation and emission when focusing at a depth of 4 mm there is considerable radial convolution and the bars of 0.25 mm width are not well resolved. Sharper representation of each edge can be obtained at smaller focal depths, such as the 2 mm data shown in blue and magenta in Fig 7. Other than this consideration, the analysis of the scan data from the artifact can proceed identically to those of absorbance intensity data. For the data shown in Fig 7, a radial correction factor of 0.9979 was obtained, with a rotation of 2.30°.

## Discussion

The use of the lithographically patterned window allows accurate external calibration of the radial scale of the most common AUC detectors with an estimated relative uncertainty of ≈0.2 %. The residual uncertainty in the radial calibration is dominated by limitations in radial resolution and the lack of detailed control in the optical detection configuration in the commercial instruments used here. The results from the artifact are more than an order of magnitude better than the experimentally observed errors from standard instrument calibration. As described previously, a large-scale study found radial magnification errors to be ≈1 % ± 3 % (mean and standard deviation), with individual instruments having reproducible errors in excess of 15 % [25]. Potential causes for these errors are varied, including incorrectly assembled counterbalance masks, user mis-assignment of calibration landmarks in the interference optics, and subtle malfunctions of instrument parts such as the stepping motor assembly of the absorbance system. As shown in the multilaboratory study, independent, external verification and correction of the radial scale are indispensable. After calibration using the lithographically patterned window, the residual error, when propagated to sedimentation coefficients, is now smaller than the contributed errors from other sources. These include errors from rotor temperature and solvent viscosity uncertainty, which is ≈0.5 % with a temperature error of 0.2 °C after iButton calibration [2, 22]. The radial calibration artifact can thus support further instrument improvements without being the limiting factor for calibration.

Additionally, an application not examined in the present work is the detection and correction of non-linear distortions in the radial dimension, which were observed in a significant fraction of instruments [25]. These cannot be addressed with the two-point or single-point standard calibrations of commercial detectors. However, due to the availability of many reference points in the lithographically patterned artifact presented here, second- or third-order distortions should be recognizable even better than from the previously used steel mask [25]. Instead of the simple application of a calibration correction factor, in this case a back-transform of the measured data into an accurate linear scale is possible [25].

A further benefit of periodically carrying out the external radial calibration is the detection of changes in the optical systems, for example, in the radial resolution or even in the absolute position of the edges if the calibration window is kept permanently assembled with the other cell components. In our experience, we have found this highly useful for ensuring consistency and troubleshooting of AUC detection.

The external radial magnification calibration is intended to be used in conjunction with corresponding external calibrations in temperature, scan time corrections, and further verified by measuring the known sedimentation coefficient of a reference molecule [2, 21, 25]. This provides a test for consistency, which will flag any calibration errors. In our laboratory, we routinely carry out such control experiments every few months as well as after instrument maintenance and repairs, to ensure accuracy of the results and to monitor for sudden unexpected changes, which are known to occur periodically for unknown reasons, as well as for possible slow deterioration of instrument calibration. The availability of calibration windows as NIST SRMs and the software we have released will facilitate the implementation of external calibration as part of a routine protocol in AUC.

## Supporting information

S1 Dataset Archive with raw scan data from different detection systems. (ZIP)
